# Proportionality in Healthcare Governance: A Conceptual Review

**DOI:** 10.7759/cureus.100577

**Published:** 2026-01-01

**Authors:** Stylianos Papalexandris, Thomas Fotiadis, Lamprini Seremeti, Dimitrios Folinas, Sofia D Anastasiadou

**Affiliations:** 1 Department of Production and Management Engineering, Democritus University of Thrace, Xanthi, GRC; 2 Department of Management and Production Engineering, Democritus University of Thrace, Xanthi, GRC; 3 Department of Regional and Economic Development, Agricultural University of Athens, Amfissa, GRC; 4 Department of Supply Chain Management, International Hellenic University, Katerini, GRC; 5 School of Economics, Administration and Computer Science, Neapolis University Pafos, Pafos, CYP; 6 Department of Midwifery, University of Western Macedonia, Ptolemaida, GRC

**Keywords:** decision-making in healthcare, emotional intelligence, healthcare governance, healthcare leadership, principle of proportionality

## Abstract

Healthcare governance requires leadership that balances organizational efficiency, staff well-being, and patient-centered care. Leadership decisions often involve dilemmas where competing values and uncertainty render decision-making implicit and opaque. Current leadership models provide limited guidance on how such decisions can be made ethically accountable. This conceptual paper introduces the principle of proportionality as a normative framework for healthcare leadership. Drawing on its established role in legal and ethical reasoning, the three-stage proportionality test, suitability, necessity, and balance, is adapted to guide leadership choices in organizational and clinical contexts. Applying proportionality to leadership enables implicit judgments to be reframed as explicit reasoning processes. The suitability stage ensures that measures are aligned with intended objectives; the necessity stage requires consideration of available alternatives; and the balance stage evaluates whether the burdens imposed are proportionate to the benefits achieved. This structured approach strengthens the ethical justification of leadership actions, enhances transparency, and mitigates arbitrariness. It also contributes to trust-building among staff, patients, and the wider public by demonstrating that trade-offs are navigated with fairness, necessity, and rational balance. Leadership guided by proportionality offers a novel conceptual contribution to healthcare governance. By embedding structured ethical reasoning into decision-making, accountability and legitimacy are promoted in contexts of uncertainty and competing demands. The proposed framework provides a foundation for further empirical investigation and offers practical implications for leadership training, organizational policy, and the development of ethically robust healthcare governance models.

## Introduction and background

Healthcare organizations operate in high-risk environments characterized by uncertainty, resource constraints, and competing operational and ethical priorities. Leaders in these settings must continually navigate tensions between conflicting goals, including patient safety, equity and access, organizational efficiency and sustainability, workforce well-being, and innovation. Within this complex landscape, leadership has long been recognized as a decisive factor in shaping organizational culture, improving patient outcomes, and guiding teams through volatile and rapidly evolving conditions [1]. Over the years, an extensive range of leadership styles has been examined in the healthcare literature, including transformational, transactional, authentic, clinical, opinion-based, participative, distributed, responsible, digital, patient-centered, charismatic, authoritarian, and laissez-faire approaches, each offering distinct advantages and limitations within specific contexts [2,3]. Despite this breadth, no single leadership model has demonstrated universal effectiveness in addressing the multidimensional challenges inherent to healthcare systems. Consequently, a persistent gap remains between leadership theory and practice.

In practice, healthcare leadership frequently involves implicit trade-offs between legitimate yet competing values (e.g., safety vs. timeliness; autonomy vs. equity; staff well-being vs. throughput). Nevertheless, existing leadership theories do not provide a principled and systematic framework for articulating or justifying these trade-offs across diverse scenarios. This limitation underscores the need for more context-sensitive and ethically grounded leadership models capable of guiding decision-making in complex clinical and organizational environments. For instance, the following scenario illustrates why healthcare leadership is not only about choosing a “style,” but also about making and justifying value-based trade-offs under pressure. At 2:00 a.m., the emergency department is overcrowded, beds are limited, and two nurses have called in sick. A senior clinician insists on admitting a frail patient “just in case,” while the bed manager argues that reserving capacity for imminent trauma arrivals is safer for the broader patient population. Meanwhile, staff exhaustion is rising, and delays in triage are already affecting waiting times for other patients. The leader must decide quickly, yet whatever decision is taken will predictably burden some patients and staff while benefiting others.

Addressing this gap, recent scholarship increasingly highlights emotional intelligence as a “silver thread” running through effective leadership across healthcare settings [4]. Emotional intelligence has been linked to core competencies such as trust building, high-quality communication, conflict resolution, staff engagement, resilience, and patient-centered care [5]. However, its “stricto sensu” integration into existing leadership models remains largely implicit, inconsistent, or incomplete. Emotional intelligence is difficult to measure empirically, varies considerably across cultures and organizational structures, and has yet to be systematically incorporated into formal leadership frameworks or training programs. As a result, while it may help explain how leaders mobilize individuals and teams ethically and sustainably, it does not provide guidance on how to weigh competing values when they collide, nor does it offer a defensible rationale for communicating and justifying such decisions to stakeholders or oversight bodies.

Accordingly, a substantive gap persists across healthcare leadership literature concerning the translation of soft-skill competencies into structured decision-making frameworks that balance organizational efficiency with ethically sound care. A promising bridge lies in the legal principle of proportionality-a normative standard requiring the balanced evaluation of competing interests and the selection of the least restrictive, most effective means to achieve a legitimate objective [6]. Reinterpreted within a healthcare leadership context, proportionality can complement emotional intelligence by offering a transparent, auditable rationale for decision-making among feasible alternatives. It requires that any chosen action be suitable (i.e., advancing a legitimate clinical or organizational aim), necessary (i.e., no less-restrictive option would achieve comparable outcomes), and proportionate in the strict sense (i.e., benefits to patients, staff, and organizational performance outweigh associated burdens or rights restrictions). Integrating emotional intelligence with proportionality thus opens a pathway toward a leadership paradigm that is simultaneously compassionate, accountable, and context-sensitive.

The methodology employed in this article follows the conventions of a conceptual paper. Rather than systematically reviewing, mapping, or synthesizing existing evidence, the aim is to introduce and theoretically justify the application of a legal principle to the domain of healthcare leadership. Whereas systematic reviews, scoping reviews, and mapping studies focus on empirical aggregation and evidence synthesis [7], conceptual papers seek to develop, adapt, and extend theoretical constructs across disciplinary boundaries. In this work, the principle of proportionality-rooted primarily in constitutional and labor law-is reconceptualized as a foundational element for ethical and effective decision-making in healthcare governance. Through argumentative reasoning and comparative analysis with established leadership models, this paper proposes a new theoretical framework that clarifies how leaders can make defensible value-based decisions in complex healthcare settings. Conceptual papers of this nature are widely recognized as essential contributions to innovation, interdisciplinary integration, and the advancement of scientific knowledge [8].

The contribution of this paper is twofold. First, through a critical examination of contemporary healthcare leadership theory, it demonstrates that while emotional intelligence has emerged as a pervasive and unifying feature across leadership models, it remains insufficient as a standalone foundation for accountable decision-making. Second, it introduces the principle of proportionality as a rule-governed framework capable of addressing this limitation. The paper begins by examining prevailing leadership styles and the prominent role of emotional intelligence, highlighting both its strengths and its conceptual and practical limitations. It then advances proportionality as a bridging mechanism that enables the development of a novel leadership style for healthcare, one that forms the central contribution of this study.

## Review

Debate on the best healthcare leadership style

Scope of the Review

Given the conceptual nature of this article, the literature discussed is intentionally selective rather than exhaustive; that is, sources are chosen for their representative coverage of dominant healthcare leadership styles and for their explanatory leverage in supporting the central argument about value-based trade-offs. Accordingly, references are used with claim-specific intent, distinguishing evidence syntheses from illustrative empirical studies and conceptual contributions, to avoid treating the leadership field as monolithic. Likewise, proportionality scholarship is invoked primarily in its normative-legal sense, as a structured discipline of justification (suitability, necessity, and proportionality stricto sensu), and is explicitly connected in this paper to recurrent healthcare leadership decisions (e.g., staffing redeployment, service prioritization, and restrictions affecting staff autonomy and patient access). Broader philosophical or engineering uses of “proportionality” are included only as brief contextual background and are not relied upon as the basis for the proposed healthcare leadership model.

Diversity of Healthcare Leadership Styles and the “No Single Best Model” Problem

The question of which leadership style is most effective in healthcare has been examined extensively [9-11], yet a definitive answer remains elusive [12-14]. A substantial body of literature reflects strong academic and practical interest in understanding healthcare leadership; however, this abundance of research also contributes to fragmentation and divergent interpretations. Rather than converging on a unified model, studies frequently highlight different leadership qualities depending on the context of inquiry, the methodological approach, and the disciplinary perspective underpinning the research.

Leadership Qualities Mapped to Dominant Styles

A considerable strand of literature emphasizes the alignment between particular leadership qualities and corresponding styles. For instance, studies focusing on transformational leadership highlight visionary capacity and the ability to articulate a shared mission as core attributes [15,16]. Other research underscores empathy and compassion as hallmarks of servant leadership [17], while decisiveness, authority, and adherence to structure are commonly associated with transactional [18] and authoritarian leadership [19]. Ethical leadership foregrounds principles of fairness, integrity, and moral responsibility, whereas clinical leadership emphasizes professional expertise and evidence-based decision-making [20]. More contemporary models, such as distributed [21] and patient-centered leadership [22], prioritize empowerment, collaboration, and shared decision-making, reflecting a broader shift from hierarchical to relational paradigms.

Leadership Performance Across Healthcare Contexts and Operational Conditions

A parallel segment of the literature investigates the performance of leadership styles across specific healthcare contexts. Transactional leadership, for example, has been shown to support operational stability and accountability, whereas autocratic leadership can prove effective in high-pressure or emergency situations where rapid decision-making is required [23]. Meanwhile, managerial perspectives tend to emphasize efficiency, performance metrics, and quality outcomes [24], while relational and humanistic perspectives focus on staff well-being, trust building, and ethical responsibility [25]. Opinion leadership, drawing from sociological theory, highlights the influence of informal networks, credibility, and peer recognition over formal authority structures [26].

Methodological Trends and Heterogeneity in Healthcare Leadership Research

Methodologically, research on healthcare leadership is dominated by qualitative and quantitative studies, as well as thematic analyses that explore experiences and perceptions across clinical settings. Systematic reviews and meta-analyses [27-29] have attempted to synthesize this evidence, yet the underlying methodological heterogeneity often complicates direct comparisons. Qualitative studies [30-34] offer nuanced insights into how nurses, physicians, and managers experience leadership, frequently emphasizing relational dimensions such as trust, empathy, and collaboration. These findings, however, tend to be context-specific. Quantitative studies [35-37], by contrast, aim to measure the impact of leadership styles on outcomes such as job satisfaction, staff turnover, patient safety, and quality of care. While these studies establish important correlations, they often lack the explanatory depth needed to illuminate underlying mechanisms.

Emotional Intelligence as a Cross-Cutting Competency in Healthcare Leadership

Amid this diversity of leadership models, a growing body of research specifically examines emotional intelligence as a determinant of leadership effectiveness [38,39]. Numerous studies identify emotional intelligence as a critical competency enabling leaders to manage stress, build trust, and foster resilience within healthcare teams [40]. Leaders with high emotional intelligence are better positioned to navigate the emotional demands of healthcare, including staff burnout, patient anxiety, and ethical dilemmas [41]. Evidence increasingly suggests that emotional intelligence functions not as an auxiliary trait but as a central factor shaping the effectiveness of transformative, servant, participative, and other relational leadership approaches [42]. This emphasis reflects a broader shift within healthcare leadership scholarship toward relational and human-centered models [43,44].

Synthesis: Why Leadership Trade-Offs Remain Under-Justified

Taken together, the literature on healthcare leadership styles reveals a field that is both conceptually rich and empirically fragmented (see Table 1). Studies highlight a wide array of leadership qualities (e.g., vision, empathy, integrity, expertise), applied across diverse clinical settings (e.g., intensive care units, outpatient clinics), interpreted through varying theoretical lenses (e.g., managerial, relational, ethical, emotional), and examined using heterogeneous methodologies (e.g., qualitative, quantitative, mixed methods). This plurality demonstrates that leadership in healthcare is inherently context-dependent and multidimensional, making the identification of a single “best” style neither feasible nor theoretically justified. Instead, the evidence points toward the need for integrative frameworks that combine effective elements across leadership styles while also offering normative guidance for resolving competing priorities.

**Table 1:** Comparative table of healthcare leadership styles. ^a^ The leadership styles in healthcare are listed in alphabetical order. Table created by the authors.

Leadership Style^a^	Strengths	Limitations
Authentic	Builds trust through transparency and integrity; encourages open communication and collaboration; promotes ethical decision-making; improves staff morale and patient-centered culture	Requires high self-awareness and emotional maturity; may be perceived as slow in urgent decision-making; difficult to maintain authenticity under organizational pressure
Autocratic or Authoritarian	Quick decision-making, vital in crises or emergencies; clear direction and expectations; effective in hierarchical or military-like healthcare structures	Can suppress creativity and innovation; reduces staff motivation and morale; risk of high turnover due to lack of empowerment
Ethical	Promotes fairness, integrity, and justice in patient care; enhances trust between patients, staff, and leadership; supports compliance with healthcare regulations and ethics	May conflict with financial or organizational pressures; can be considered idealistic; in resource-constrained systems; requires strong ethical frameworks across the team
Charismatic	Inspires enthusiasm and motivation among staff; creates a shared vision and strong organizational culture; can unite teams during times of change or crisis	Risk of over-dependence on leader’s personality; vision may overshadow operational details; succession planning challenges if leader departs
Clinical	Direct influence on bedside care and patient outcomes; strengthens evidence-based practice; encourages professional role modeling for healthcare staff	Scope often limited to clinical environments; may lack influence in broader organizational strategy; time demands may conflict with clinical duties
Digital	Drives adoption of healthcare technologies; encourages innovation and modernization; prepares organizations for digital transformation	Resistance from staff lacking digital skills; may prioritize technology over human-centered care; rapid tech changes require continuous adaptation
Laisser-Faire	Encourages autonomy for highly skilled professionals; fosters innovation and independent thinking; can work well in research and academic healthcare settings	Lack of guidance may cause confusion and inefficiency; risk of poor accountability and team dysfunction; not suited for high-stakes clinical environments
Distributed or Shared	Promotes teamwork and shared responsibility; enhances decision-making with diverse input; builds resilience in multidisciplinary healthcare teams	Risk of blurred accountability and conflict; slower decisions in urgent situations; requires strong communication structures
Opinion	Influences practice change through credibility and expertise; acts as a role model within professional groups; encourages adoption of innovations in healthcare	Influence may be limited to specific specialties; risk of bias if opinions lack evidence base; may not have formal authority to enforce changes
Participative or Democratic	Encourages collaboration and shared decision-making; boosts morale standards and team cohesion; enhances commitment to decisions among staff	Slower decision-making; risk of conflicts; less effective in urgent or crisis settings
Patient-centered	Focuses on quality, safety, and patient experience; improves trust and satisfaction among patients and families; aligns leadership with core healthcare mission	May collide with organizational financial goals; resource-intensive to implement effectively; requires continuous feedback and adaptation
Responsible	Balances economic, social, and environmental responsibilities; encourages accountability and sustainability; build stakeholder trust beyond patients	May face resistance from profit-driven organizations; hard to measure responsibility outcomes; can be complex in fragmented healthcare systems
Resonant	Builds strong, emotionally intelligent relationships; improves staff engagement and well-being; reduces workplace stress and burnout	Requires high emotional intelligence and empathy; time-intensive, not always practical in emergencies; risk of emotional exhaustion for leaders
Servant	Puts patients and staff needs first; builds trust, empathy, and strong ethical culture; improves job satisfaction and retention	Decision-making may be slowed by consensus focus; risk of leader being exploited; may lack assertiveness in conflict situations
Situational	Flexible, adapts to staff competence and readiness; effective in diverse teams with varying skill levels; balances direction and support	Requires accurate judgment of staff abilities; time-consuming to adapt style continuously; risk of inconsistency if poorly executed
Transformational	Inspires innovation and continuous improvement; builds strong organizational vision and motivation; strong focus on change management and patient safety	Can lead to burnout due to high expectations; may overlook short-term operational issues; risk of over-reliance on leader’s vision

Bridge to the Proposed Framework: Proportionality as a Rule-Governed Justification Tool

In response to this gap, the present paper proposes a novel leadership model grounded in the principle of proportionality. This approach synthesizes vision, emotional intelligence, and structured ethical reasoning into a unified framework capable of guiding decision-making in complex healthcare environments.

According to Table 1, most healthcare leadership styles share the overarching goals of improving patient outcomes, enhancing team performance, and optimize organizational efficacy. However, they differ markedly in their approaches to authority, decision-making, and interpersonal dynamics. Autocratic and transactional leadership styles, for example, emphasize control, compliance, and operational efficiency. In contrast, participative, servant, and distributed leadership prioritize collaboration, empowerment, and shared responsibility. More contemporary approaches, including authentic, resonant, ethical, and transformational leadership, place strong emphasis on values, interpersonal relationships, and the leader’s personal influence, reflecting the growing recognition of emotional intelligence as a critical component of effective leadership.

Despite the consistent emphasis on emotional intelligence across the literature, several challenges remain unresolved, including difficulties in measurement, gaps in formal training, and variability in its impact across cultural and clinical contexts. Addressing these limitations requires a complementary framework capable of providing structured, ethically grounded guidance for decision-making. In this regard, the principle of proportionality, widely applied in constitutional law, labor law, and international humanitarian law, offers a promising integrative tool. Its incorporation into healthcare leadership theory has the potential to bridge existing gaps by introducing a transparent, rule-governed method for balancing competing values and justifying leadership decisions within complex healthcare environments.

Proportional healthcare leadership style

Healthcare institutions are inherently hierarchical and highly regulated, with leadership teams making decisions that affect a wide array of stakeholders, including patients, nurses, physicians, support staff, and the general public [45]. These decisions frequently involve mutually incompatible ethical and operational considerations, for example, balancing patient safety with staff autonomy, or reconciling financial constraints with equitable access to care [46]. Despite the extensive range of leadership styles that inform decision-making in healthcare, no established standard exists for determining whether a leader’s decision is proportionately justified relative to its anticipated impact. This paper argues that the principle of proportionality, adopted from legal theory and judicial practice, provides a robust framework for ensuring that leadership decisions in healthcare are both operationally effective and ethically defensible.

The principle of proportionality is a foundational evaluative tool in public and constitutional law, used to assess whether state action is justified in relation to its aims. It has also been applied successfully in employment and labor law to balance power asymmetries and protect individual rights [47]. Proportionality is not novel to the healthcare sector: it has been widely invoked during the COVID-19 pandemic to assess the legitimacy of public health interventions such as lockdowns, mandatory vaccinations, and mobility restrictions [48-50]. These studies primarily evaluate policy measures after their implementation, focusing on whether the chosen interventions were justified in relation to their legal, social, and clinical consequences. What distinguishes the present study is that it does not treat proportionality merely as a legal doctrine for post hoc policy evaluation, but instead conceptualizes it as a proactive and systematic model for healthcare leadership decision-making.

Therefore, this paper proposes the structured adoption of proportionality within healthcare leadership, a domain characterized by complex ethical dilemmas, hierarchical dynamics, and high-risk operational processes. By adapting the established three-stage proportionality test to organizational governance, healthcare leaders can enhance fairness, transparency, and ethical rigor in their decisions. Such an approach is necessary to preserve institutional integrity, uphold professional morality, and maintain a consistent commitment to patient-centered care.

The subsections that follow are designed to build a clear and cohesive justification for integrating proportionality into healthcare leadership. First, the concept of proportionality is defined within its legal context, followed by a detailed analysis of its three-stage evaluative test, illustrated through an original hypothetical example. Next, the proposed proportionality-based leadership model is compared with the most representative existing leadership styles in healthcare to highlight its distinctive ethical orientation and structured approach to governance. Finally, the paper presents both supporting arguments and principled critiques to offer a balanced assessment of the model’s strengths and limitations, thus clarifying its conceptual foundation and articulating the basis for its ethical defensibility.

Conceptualization of the Principle of Proportionality

Proportionality, as a cross-disciplinary concept, denotes a balanced correspondence between means and ends [51]. Its meaning varies across intellectual traditions. In the social and philosophical context, it is closely associated with Aristotle’s “Golden Mean” [52], while in engineering it is used to describe the harmony between form and function [53]. From an ethical perspective, proportionality serves as a rule of justification for decisions made under uncertainty or in environments characterized by conflicting values [54]. Embedded into legal doctrine, proportionality has transformed the exercise of public and private authority from discretionary action into a practice of structured and reasoned judgment [55].

As a discipline of justification, the principle of proportionality requires that any interference with protected interests, such as individual rights, autonomy, or welfare, must be appropriately tailored to a legitimate aim and must not exceed what is necessary to achieve that aim [56]. Originating in continental European constitutional jurisprudence and later integrated into supranational legal systems, proportionality operates as a meta-principle that organizes reasoning and constrains decision-makers across a wide range of contexts [57]. Conceptually, it mediates the tension between collective objectives and individual rights by requiring decision-makers to demonstrate a transparent and rational commitment to both [58].

Within healthcare leadership, proportionality provides a structured and principled framework for navigating the ethical complexities inherent in everyday decision-making. It offers a disciplined method through which leaders can justify their actions in a manner that is transparent, accountable, and normatively coherent.

The Three-Stage Proportionality Test

Healthcare leadership is characterized by complex, often time-pressured decisions in which competing interests, such as clinical outcomes, staff well-being, operational efficiency, and ethical responsibilities, must be continually balanced [59]. This dynamic mirrors challenges observed in employment and labor contexts, where the principle of proportionality is applied to prevent excessive or unjustified uses of managerial authority [60]. Interpreting proportionality within healthcare settings offers a structured ethical framework for evaluating whether leadership decisions are not only effective, but also fair, justified, and necessary.

Drawing on the proportionality framework widely used in constitutional and labor law, healthcare leadership can adopt a similar three-stage test encompassing the criteria of suitability, necessity, and proportionality in the strict sense [61]. First, a decision must be suitable, meaning that there is a rational connection between the action taken and the legitimate objective pursued, such as improving patient safety or reducing clinical risk. Second, it must be necessary, indicating that no less intrusive or burdensome alternative is available to achieve the same outcome. Third, the action must be proportionate in the strict sense, requiring that the anticipated benefits outweigh any adverse effects imposed on staff, patients, or institutional values.

Consider, for example, a healthcare institution experiencing chronic staff shortages that implements a mandatory redeployment policy, temporarily transferring nurses from outpatient clinics to high-acuity inpatient units. At the stage of suitability, such a measure is logically connected to the legitimate goal of maintaining safe staffing levels in critical areas and ensuring clinical continuity. Regarding necessity, leadership must assess whether less disruptive alternatives, such as voluntary redistribution with incentives, short-term external staffing, or modified scheduling, could achieve similar results. If these options have been exhausted or shown insufficient, mandatory redeployment may meet the necessity threshold. At the final stage of proportionality in the strict sense, leaders must weigh improvements in patient safety and the alleviation of workload in high-intensity units against the potential stress, skill mismatch, or operational disruption experienced by redeployed staff and outpatient services. If the benefits to patient care and workforce sustainability outweigh the temporary burdens imposed elsewhere, the policy may be ethically justified as proportionate. This example illustrates how the three-stage test guides action and facilitates fair and transparent decision-making.

Comparison with Dominant Styles

Comparing the proposed proportional leadership model with widely adopted leadership styles, such as transactional, transformational, and autocratic approaches, highlights its distinctive contributions and practical positioning within existing frameworks.

Unlike transactional leadership, which relies on reward-and-punishment mechanisms to ensure performance compliance [62], proportional leadership focuses on the moral justification, necessity, and balanced impact of decisions. Transactional leadership is effective in routine, protocol-driven environments but tends to overlook ethical tensions inherent in complex healthcare settings [63]. In contrast, proportional leadership provides a structured decision-making process, centered on suitability, necessity, and proportionality in the strict sense, that can accommodate both ethical and clinical complexities while fostering institutional trust.

Relative to transformational leadership, which seeks to inspire teams through vision, motivation, and intellectual stimulation [64], proportional leadership offers a more grounded and accountable approach. While transformational leaders may pursue ambitious change, unintended consequences may arise if initiatives are not subjected to systematic ethical scrutiny [65]. Proportional leadership mitigates this risk by requiring reasoned justification and equity, ensuring that transformational goals remain ethically sound and socially responsible.

In comparison with autocratic leadership, where decisions are made unilaterally and stakeholder input is limited [66], proportional leadership represents a more humane and participatory alternative. Autocratic approaches may deliver rapid decisions but often undermine long-term staff well-being and morale [67]. Proportional leadership, by contrast, emphasizes transparency, fairness, and the balanced distribution of burdens, fostering institutional cohesion and psychological safety.

This focused comparison underscores the need to introduce proportionality as a guiding mechanism within healthcare leadership. By explicitly addressing operational, ethical, and relational considerations, proportional leadership serves as a pivotal bridge between efficiency and ethical responsibility, thereby supporting more accountable and defensible decision-making.

Arguments in Favor of a Proportionality-Based Healthcare Leadership Model

The introduction of a new leadership model grounded in the principle of proportionality requires a clear justification of its added value. The following subsections outline the primary arguments supporting its adoption within healthcare, particularly given the growing demand for leadership that is ethically robust, transparent, and context-sensitive. Unlike conventional models that prioritize efficiency, compliance, or inspiration, proportional leadership offers a structured ethical framework that ensures leadership actions are effective, fair, necessary, and balanced in their impact.

First, proportional leadership enhances the ethical legitimacy of decision-making. The proportionality principle ensures that actions are suitable, appropriate, and necessary, criteria that align with foundational bioethical values such as justice, beneficence, and non-maleficence. In a field in which decisions routinely affect human well-being and rights, leadership must be more than operationally competent; it must also be morally defensible. Proportional leadership compels leaders to justify decisions not only in terms of outcomes, but also with respect to fairness, necessity, and justification. This strengthens ethical accountability and enhances institutional credibility.

Second, proportional leadership builds trust among staff, patients, and other stakeholders by emphasizing balanced and minimally harmful decision-making. When leaders can demonstrate that a policy was chosen because it represents the least burdensome and most appropriate option available, they are more likely to secure acceptance and reduce resistance. Trust is particularly critical in high-stakes or resource-constrained environments, where adherence to protocols and psychological resilience are essential. Proportionality thus provides a foundation for open dialogue, shared reasoning, and cooperative culture.

Third, proportional leadership fills a notable gap in existing models. Transactional and autocratic styles prioritize control and operational performance, often at the expense of ethical deliberation. Even transformational leadership, while relational and empowering, lacks a structured mechanism for evaluating potential harms or trade-offs. Proportional leadership embeds a systematic ethical assessment into the decision-making process, preventing excess, encouraging alternatives, and promoting self-restraint in the exercise of institutional authority. These position proportionality not as an ancillary ethical consideration, but as a corrective mechanism within leadership praxis.

Fourth, proportional leadership supports adaptability in dynamic and uncertain healthcare environments. Because the proportionality test is both structured and flexible, it allows leaders to reassess decisions as circumstances evolve. This adaptability is essential during crises, such as pandemics, supply shortages, or clinical triage dilemmas, where leaders must act quickly while upholding ethical standards. Rather than relying solely on hierarchical authority or improvisation, proportional leadership enables context-sensitive yet principled responses.

Finally, proportional leadership contributes to greater equity by requiring explicit consideration of how burdens and benefits are distributed across groups. Before implementing measures such as extended shifts or service reductions, proportional leaders consider whether certain departments, professional groups, or patient populations are disproportionately affected. In doing so, proportionality promotes fairness, reduces structural bias, and encourages more inclusive decision-making.

Arguments Against a Proportionality-Based Healthcare Leadership Model

Engaging critically with potential objections strengthens the conceptual robustness of the proposed leadership model. Addressing counterarguments demonstrates intellectual rigor, enhances research quality, encourages innovation, and ensures a comprehensive analysis of the topic [68].

A central critique concerns the time and deliberation required to apply structured ethical reasoning. The three-stage proportionality test, assessing suitability, necessity, and proportionality in the strict sense, requires reflection, information gathering, and sometimes consultation. In emergency scenarios such as trauma response, epidemic control, or acute workforce shortages, leaders often must act rapidly. Critics argue that incorporating deliberative proportional analysis may slow decision-making, create procedural bottlenecks, or frustrate frontline staff who require clear and immediate direction.

Another concern relates to leaders’ varying levels of training in ethical reasoning and legal analysis. Effective implementation of proportionality depends on consistent and competent application. However, many healthcare leaders have limited formal training in normative ethics or legal justification. This gap raises the risk of inconsistent, overly subjective, or superficial application of the model. Without adequate educational support, leaders may fail to use proportionality correctly or may abandon it under pressure.

A further challenge lies in the inherent subjectivity of weighing competing values. Determining what is “necessary” or “strictly proportionate” can vary across stakeholders. Hospital administrators may view restricting elective services as proportionate during financial constraints, whereas clinicians may consider such measures ethically problematic. This variability may lead to stakeholder conflict, tension, or even policy gridlock.

There is also a risk that proportionality, if applied uncritically, could inadvertently normalize systemic deficiencies. In chronically underfunded systems, leaders may repeatedly justify restrictive or burdensome policies as proportionate responses to scarcity, thereby shifting attention away from structural reform. Over time, proportionality could become a mechanism for legitimizing suboptimal conditions rather than challenging them.

Finally, organizational culture may pose barriers. In highly hierarchical or authoritarian healthcare systems, proportional leadership may be resisted by those who view ethical deliberation as a threat to efficiency or authority. Leaders operating in such environments may lack autonomy to apply proportional reasoning, limiting the feasibility of implementation and reinforcing skepticism toward the model.

Discussion

The absence of a definitive justification for any single “best” healthcare leadership style reflects the inherent complexity and heterogeneity of the healthcare sector. Although scholars have identified numerous leadership approaches and empirical evidence consistently links emotional intelligence to effective leadership outcomes, emotional intelligence alone is not prescriptive. It represents an important category of interpersonal and regulatory skills, but it does not offer a systematic framework for navigating competing institutional demands or for justifying decisions in ethically challenging contexts.

Proposing a healthcare leadership model grounded in the principle of proportionality is therefore essential in today’s ethically complex, resource-constrained, and high-pressure medical environments. Above all, proportionality provides a structured, rule-governed framework for decision-making that surpasses intuition, authority, or ad hoc reasoning. Healthcare leaders routinely make high-stakes decisions regarding staffing, resource allocation, clinical priorities, and organizational strategy, decisions that involve competing rights, interests, and risks. A proportionality-based model ensures that such choices are not only operationally effective but also morally justified, thereby enhancing the legitimacy and credibility of leadership actions among staff, patients, and stakeholders.

Furthermore, proportional leadership mitigates the risk of managerial overreach and protects staff well-being. Traditional top-down models may inadvertently produce excessive burdens on frontline workers, contributing to burnout, disengagement, or resistance. Proportional leadership, by contrast, compels leaders to evaluate whether a directive constitutes the least harmful and least intrusive means of pursuing its intended goal and whether its expected benefits sufficiently outweigh its costs. This approach respects professional autonomy and contributes to psychological safety, two factors empirically associated with improved clinical performance, higher team cohesion, and reduced turnover. Leaders who transparently justify their decisions are more likely to earn trust and foster cooperation, particularly during crises.

In addition, proportionality inherently promotes equity and inclusivity within healthcare organizations. It obliges leaders to assess the differentiated impact of policies across diverse professional and demographic groups, including nurses, junior physicians, administrative personnel, and individuals from underrepresented backgrounds. For instance, applying proportionality would require leaders to consider whether a uniform policy, such as a blanket restriction on leave, disproportionately affects caregivers, part-time workers, or marginalized groups. Institutionalizing such reflection fosters a fairer organizational culture in which justice, respect, and distributive equity are explicitly integrated into leadership practices.

Finally, proportionality aligns closely with the foundational ethical principles of medicine-justice, beneficence, non-maleficence, and respect for autonomy. Embedding this legal-ethical principle into leadership practice helps bridge the persistent gap between administrative decision-making and clinical ethics. By enabling leaders to articulate decisions in terms of necessity, justification, and balance, proportional leadership strengthens the moral coherence of healthcare governance. As the healthcare sector grows increasingly complex, transparent, and morally scrutinized, a proportionality-based leadership model offers a timely and necessary evolution: one that enables leaders to remain both decisive and ethically accountable.

## Conclusions

The quest to identify the most effective healthcare leadership style has long engaged both scholars and practitioners. Although emotional intelligence consistently appears as a critical component of effective leadership, it does not sufficiently capture the complexity or ethical demands of healthcare decision-making. This paper proposes a leadership model grounded in the principle of proportionality. By applying the three-stage proportionality test, leaders can ensure decision-making that is ethical, fair, and contextually appropriate, especially in environments characterized by competing priorities and constrained resources. Future research should empirically examine the implementation of this model across diverse healthcare settings to assess and validate its practical utility, feasibility, and impact on organizational and clinical outcomes.
